# Functional Modulation of Regulatory T Cells by IL-2

**DOI:** 10.1371/journal.pone.0141864

**Published:** 2015-11-03

**Authors:** Byung-In Moon, Tae Hun Kim, Ju-Young Seoh

**Affiliations:** 1 Department of Surgery, Ewha Womans University Graduate School of Medicine, Seoul, Korea; 2 Department of Internal medicine, Ewha Womans University Graduate School of Medicine, Seoul, Korea; 3 Department of Microbiology, Ewha Womans University Graduate School of Medicine, Seoul, Korea; University of Lisbon, PORTUGAL

## Abstract

The suppressive function of regulatory T cells (Tregs) is critical to the maintenance of immune homeostasis *in vivo* and yet, the specific identification of Tregs by phenotypic markers is not perfect. Tregs were originally identified in the CD4^+^CD25^+^ fraction of T cells, but FoxP3 expression was later included as an additional marker of Tregs as FoxP3 expression was identified as being critical to the development and function of these cells. Intracellular expression of FoxP3 makes it difficult in using to isolate live and not permeabilized cells for functional assays. As such CD4^+^CD25^+^ fraction is still frequently used for functional assays of Tregs. Although, the CD4^+^CD25^+^ fraction substantially overlaps with the FoxP3^+^ fraction, the minor mismatch between CD4^+^CD25^+^ and FoxP3^+^ fractions may confound the functional characteristics of Tregs. In this study, we isolated CD4^+^FoxP3^+^ as well as CD4^+^CD25^+^ fractions from Foxp3 knock-in mice, and compared their proliferative and suppressive activity in the presence or absence of various concentrations of IL-2. Our results showed comparable patterns of proliferative and suppressive responses for both fractions, except that contrary to the CD4^+^CD25^+^ fraction the FoxP3^+^ fraction did not proliferate in an autocrine fashion even in response to a strong stimulation. In presence of exogenous IL-2, both CD4^+^CD25^+^ and CD4^+^FoxP3^+^ fractions were more sensitive than the CD4^+^CD25^-^ responder cells in proliferative responsiveness. In addition, a low dose IL-2 enhanced whereas a high dose abrogated the suppressive activities of the CD4^+^CD25^+^ and CD4^+^FoxP3^+^ fractions. These results may provide an additional understanding of the characteristics of the various fractions of isolated Tregs based on phenotype and function and the role of varying levels of exogenous IL-2 on the suppressive activity of these cells.

## Introduction

Regulatory T cells (Tregs) play a key role in the maintenance of immune homeostasis *in vivo* as they suppress almost every kind of effector cells including CD4^+^ T cells, CD8^+^ T cells, B cells, and NK cells [1,2,3,4]. Tregs comprise a minor fraction of CD4^+^ cells (~ 10% of CD4^+^ cells in the periphery) [5]. Frequency and functional activities of Tregs are modulated in various physiological and pathological processes *in vivo*, such as infection, autoimmune response and interaction with the tumor environment [5,6]. There are many studies on how Treg number is regulated, in particular in association with IL-2, but how Treg function is regulated is still remains to be under intense investigation [7,8,9,10].

Besides the functional phenotype of Tregs, as being suppressive on target cells, additional markers for these cells that provide a logical link with their suppressive function are required and have been proposed [6]. As for isolation markers of these cells, FoxP3 is a marker for these cells that has provided a high level of specificity. The intracellular expression of FoxP3, however, has hampered its utility in isolating live cells for functional assays. Several genetically engineered mice have been developed to help in this respect [11]. The CD4^+^CD25^+^ fraction has been documented to have many of the suppressive effects of candidate Tregs and studies have shown that the CD4^+^CD25^+^ fraction substantially overlaps with the FoxP3^+^ fraction; as such CD4^+^CD25^+^ fraction is still being used in many functional studies of Tregs, in particular, when the genetically engineered mice are not available or that the studies are from human subjects. However, one should be mindful of the imperfect match between the CD4^+^CD25^+^ and FoxP3^+^ fractions as investigators try to elucidate the function of the Tregs cells in various assays and that they may draw different conclusions if they use them interchangeably.

Tregs express a high level of IL-2Rα (CD25) constitutively. These cells, however, do not produce IL-2 but IL-2 is required for their proliferation, survival and function [12,13,14,15]. FoxP3, that is critical for the development and function of Tregs, while upregulating the expression of CD25, suppresses the transcription of IL-2 [16]. Not being able to make their own IL-2, but being critically dependent on exogenous IL-2 may provide a key control of these cells in terms of their proliferation and survival *in vivo*. Exogenous IL-2 is essential to the *in vitro* culture of Tregs for example and likewise, Tregs should be cultured together with cells that secrete IL-2 such as CD4^+^CD25^-^ effector T cells (Teffs) [17]. As such the *in vivo* source of IL-2 could be Teffs [18]. A dependence of Tregs on IL-2 secreted by Teffs, and suppression of Teffs that supply this IL-2 could form an elegant loop of biological feedback [7,8,19].

There are additional layers of regulation with respect to IL-2 in Tregs: Tregs inhibit the production of IL-2 in Teffs and they also deplete IL-2 from Teffs by capturing IL-2 through their highly expressed IL-2 receptors [8,20,21]. Tregs also lower the surface expression of CD25 on Teffs by inducing early receptor-shedding from Teffs [22]. In all, IL-2 concentration is critical to the fate of both Tregs and Teffs and IL-2 could be thought of providing a homeostatic control of immune responses *in vivo*.

Clinically, IL-2 has been used for immunotherapy of several kinds of cancers, such as melanoma and renal cell carcinoma [23,24]. In the meantime, recent observations showed inconsistent results of IL-2 administration; it enhanced spontaneous development of insulin-dependent diabetes in BB rats [25], turned on anergic self-reactive T cells and lead to autoimmune diseases [26]; and pharmacological dose of IL-2 treated renal cancer [24]. Low-dose IL-2, in combination with IL-21, induced anti-tumor immunity and long-term curative effects in a murine melanoma model [27]. On the other hand, opposing results showed that IL-2 administration reversed type I diabetes in NOD mice [28]; IL-2 induced oral tolerance in a murine model of autoimmunity and *in vivo* suppression by Tregs [29]. Low dose IL-2 in association with dendritic cell (DC) vaccination increased circulating Tregs in patients with metastatic renal carcinoma [30].

Thus, it is not easy to predict what happens upon IL-2 administration *in vivo*, and we investigated in the present study the proliferative and suppressive activity of Tregs in the presence of various concentrations of exogenous IL-2 *in vitro*, and compared the differences between CD4^+^CD25^+^ and CD4^+^FoxP3^+^ fractions in their functional responses to exogenous IL-2. Our data showed that in presence of exogenous IL-2, both the CD4^+^CD25^+^ and CD4^+^FoxP3^+^ fractions were more sensitive than the CD4^+^CD25^-^ responder cells in proliferative responsiveness. In addition, IL-2 had a biphasic effect on the suppressive activity of Tregs: at a relatively lose dose, IL-2 enhanced, whereas at a relatively high dose it abrogated the suppressive activity in Tregs. These observations add another layer of complexity on the molecular and cellular effects of IL-2 in these cells and may have implications about IL-2 as a means of modulating the activity of Tregs.

## Materials and Methods

### Preparation of cells

C57BL/6-*Foxp3* ^*3tm1Flv*^/J mice, that had been purchased from Jackson Laboratory (Sacramento CA, USA), were bred and kept in the animal facility of Ewha Womans University Graduate School of Medicine (Seoul, South Korea), and were used in experiments when they were 8–12 weeks old [31]. This study was performed according to the Korean Food and Drug Administration Guidelines for animal research and our protocol was specifically approved by the Institutional Animal Care and Use Committee of Ewha Womans University Graduate School of Medicine (Permit Number: 10–0133). Spleen cells were prepared by lysing erythrocytes and were first incubated with anti-mouse CD16/CD32 (2.4G2) to block their Fc receptors. Anti-mouse CD4-Pacific blue (GK1.5) and anti-CD25-APC (PC61) were added for surface staining, and CD4^+^CD25^-^ and CD4^+^CD25^+^, and CD4^+^RFP^+^ fractions were sorted by using a FACSAria^TM^ instrument (BD Biosciences, San Jose CA, USA). CD11c^+^ cells were isolated using a spleen dissociation medium (StemCell technologies, Vancouver BC, Canada), followed by density gradient centrifugation over 15.5% Accudenz (Accurate Chemical & Scientific, Westbury NY, USA), and FACS sorting after staining with anti-CD11c-PE (HL3). All the above antibodies were purchased from BD Biosciences (BD Biosciences, San Jose CA, USA). Purity of the sorted fractions was over 97%. Intranuclear staining for FoxP3 done by using a mouse regulatory T cell staining kit (eBiosciences, San Diego CA, USA) that contained the anti-FoxP3 antibody FJK-16s-PE-Cy5 showed that the RFP^+^ fraction was identical to the FoxP3^+^ fraction. Portions of CD4^+^CD25^-^ and CD4^+^CD25^+^, and RFP^+^ fractions were labeled with carboxyfluorescein diacetate succinimidyl ester (CFSE) (Invitrogen, Carlsbad CA, USA) according to protocol described elsewhere to trace the proliferation of each fraction of cells separately [32].

### *In vitro* proliferation assay with antigen-presenting cells (APCs)

In order to compare the proliferative responsiveness of CD4^+^CD25^+^, CD4^+^FoxP3^+^ fractions and CD4^+^CD25^-^ responder cells in separate cultures, 10^4^ CFSE-labeled cells were cultured in the presence of 2 x 10^3^ CD11c^+^ dendritic cells (DCs). In order to compare the proliferative responsiveness in the cocultures, 10^4^ CFSE-labeled CD4^+^CD25^+^ or CD4^+^FoxP3^+^ cells were cultured together with an equal number of unlabeled CD4^+^CD25^-^ responder cells, and reversely 10^4^ CFSE-labeled responder cells were cultured together with the equal number of unlabeled CD4^+^CD25^+^ or CD4^+^FoxP3^+^ cells, in the presence of 2 x 10^3^ DCs [33]. These cells were cultured in DMEM media supplemented with 10% FCS (Hyclone, Logan UT, USA) in a total volume of 200 μl in round-bottomed 96-well plates with various concentrations of soluble anti-CD3e alone or in combination with 100 ng/mL of soluble anti-CD28 (both mAbs from eBiosciences, San Diego CA, USA). To investigate the effects of exogenous IL-2 on the suppressive activity of CD4^+^CD25^+^ cells and CD4^+^FoxP3^+^ cells, various concentrations of recombinant mouse IL-2 (rmIL-2) (Chemicon, Temecula CA, USA) were added to the cultures of CFSE-labeled responder cells and unlabeled CD4^+^CD25^+^ or CD4^+^FoxP3^+^ cells while the cells were stimulated with 333 ng/mL of soluble anti-CD3e. After 3 days, the cells were harvested for flow cytometry.

### APC-free *in vitro* proliferation assay

In order to investigate the proliferative responsiveness of cells in an APC-free system, 10^4^ CFSE-labeled CD4^+^CD25^+^, CD4^+^FoxP3^+^ cells or CD4^+^CD25^-^ responder cells were cultured in 96-well plates where various amount of anti-CD3e were immobilized, in the presence of soluble anti-CD28 (100 ng/mL) and various concentrations of rm IL-2. After 3 days, the cells were harvested for flow cytometry.

### Flow cytometry

On the 3^rd^ day of culture, the cells were harvested and stained with anti-CD4-Pacific blue and whole cells were acquired for analysis by using WinList software (Verity, Topsham ME, USA). Precursor frequency (Pf) was estimated for cells exclusively gated for CD4^+^ live cells according to the scattering characteristics and using the “Proliferation Wizard” setting of ModFit software (Verity), as described elsewhere [34].

### Statistical analysis

Data are expressed as mean ± SE of three separate experiments. Comparison of data was performed using Kruskal–Wallis one-way analysis of variance. P values less than 0.05 were considered statistically significant.

## Results

### Overlapping distributions of CD4^+^FoxP3^+^ and CD4^+^CD25^+^ cells in splenocytes

Flow cytometric analysis of splenocytes prepared from four C57BL/6-*Foxp3* ^*3tm1Flv*^/J mice showed that 2.6 ± 0.4% of the cells were CD4^+^CD25^+^ and 2.8 ± 0.3% were CD4^+^FoxP3^+^ (n = 12) (S1 Fig). Of the CD4^+^CD25^+^ cells, 88.4 ± 3.5% were CD4^+^FoxP3^+^, while 11.5 ± 1.3% were CD4^+^FoxP3^-^. Of the CD4^+^FoxP3^+^ cells, 74.6 ± 4.2% were CD4^+^CD25^+^, while 22.5 ± 2.2% were CD4^+^CD25^-^.

### In the absence of exogenous IL-2, CD4^+^CD25^+^ and CD4^+^FoxP3^+^ cells were hypoproliferative than CD4^+^CD25^-^ responder cells in separate cultures, but hyperproliferative in the cocultures with the responder cells *in vitro*

**Fig 1:**
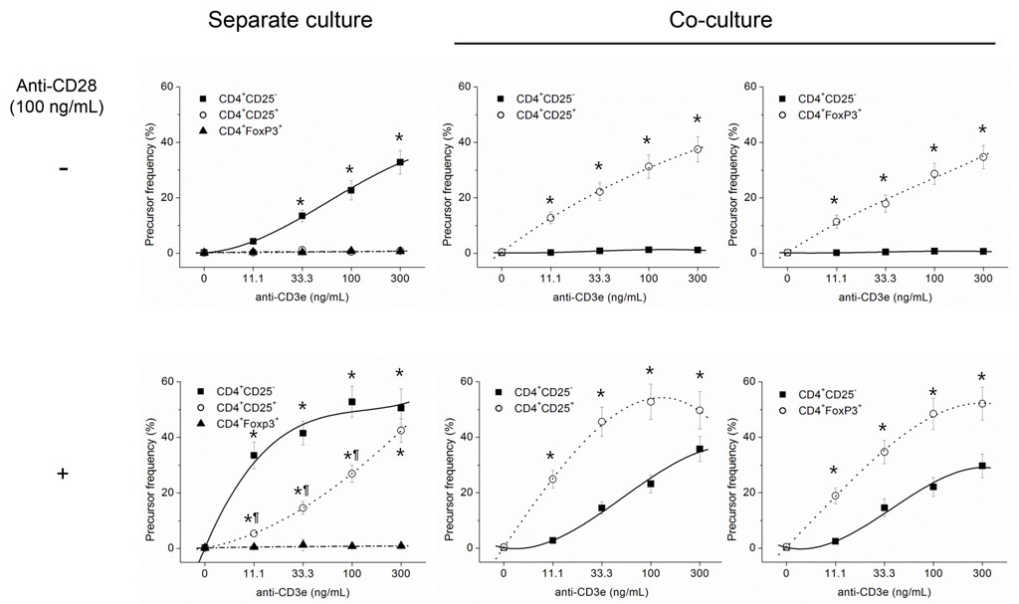
*In vitro*, CD4^+^CD25^+^ and CD4^+^FoxP3^+^ cells were hypo-proliferative compared with CD4^+^CD25^-^ responder cells in separate cultures, but hyperproliferative in the co-cultures with the responder cells. Proliferative responses of CD4^+^CD25^+^, CD4^+^FoxP3^+^ and CD4^+^CD25^-^ cells in separate (left column) or co-cultures (middle and right columns). The cells were stimulated with varying concentrations of soluble anti-CD3e alone (upper) or in combination with 100 ng/mL of soluble anti-CD28 (lower) in the presence of CD11c^+^ dendritic cells. Data are mean ± SE of three separate experiments with 4 replicate wells per dilution. *, P<0.05, between CD4^+^CD25^+^ or CD4^+^FoxP3^+^ fraction and CD4^+^CD25^-^ responder cells. ¶, P<0.05, between CD4^+^CD25^+^ and CD4^+^FoxP3^+^ fractions.

In the absence of exogenous IL-2, CD4^+^CD25^+^ and CD4^+^FoxP3^+^ cells did not proliferate at all by stimulating with soluble anti-CD3e alone (Fig 1). Addition of soluble anti-CD28 (100 ng/mL) to the stimulation with soluble anti-CD3e induced a proliferative response of CD4^+^CD25^+^ cells, but it was less than that of CD4^+^CD25^-^ responder cells. By contrast, CD4^+^FoxP3^+^ cells did not proliferate at all even in presence of anti-CD3e plus anti-CD28 stimulation. On the other hand, CD4^+^CD25^-^ responder cells proliferated well in response to soluble anti-CD3e alone as well as in combination with anti-CD28. In the co-cultures with equal number of CD4^+^CD25^-^ responder cells, stimulation with soluble anti-CD3e alone induced proliferation of CD4^+^CD25^+^ and CD4^+^FoxP3^+^ cells, but the responder cells did not proliferate at all, implying complete suppression by CD4^+^CD25^+^ or CD4^+^FoxP3^+^ cells. Addition of soluble anti-CD28 (100 ng/mL) to the stimulation with soluble anti-CD3e induced proliferation of CD4^+^CD25^-^ responder cells in the co-cultures with CD4^+^CD25^+^ or CD4^+^Foxp3^+^ cells, but the proliferative response of CD4^+^CD25^-^ responder cells was less than those of CD4^+^CD25^+^ or CD4^+^FoxP3^+^ cells. The proliferative response of the responder cells in the co-cultures was also less than that in the separate cultures again suggesting suppression by CD4^+^CD25^+^ or CD4^+^FoxP3^+^ cells.

### In the presence of exogenous IL-2, CD4^+^CD25^+^ and CD4^+^FoxP3^+^ cells were more sensitive than CD4^+^CD25^-^ responder cells in their *in vitro* proliferative response*s*

**Fig 2:**
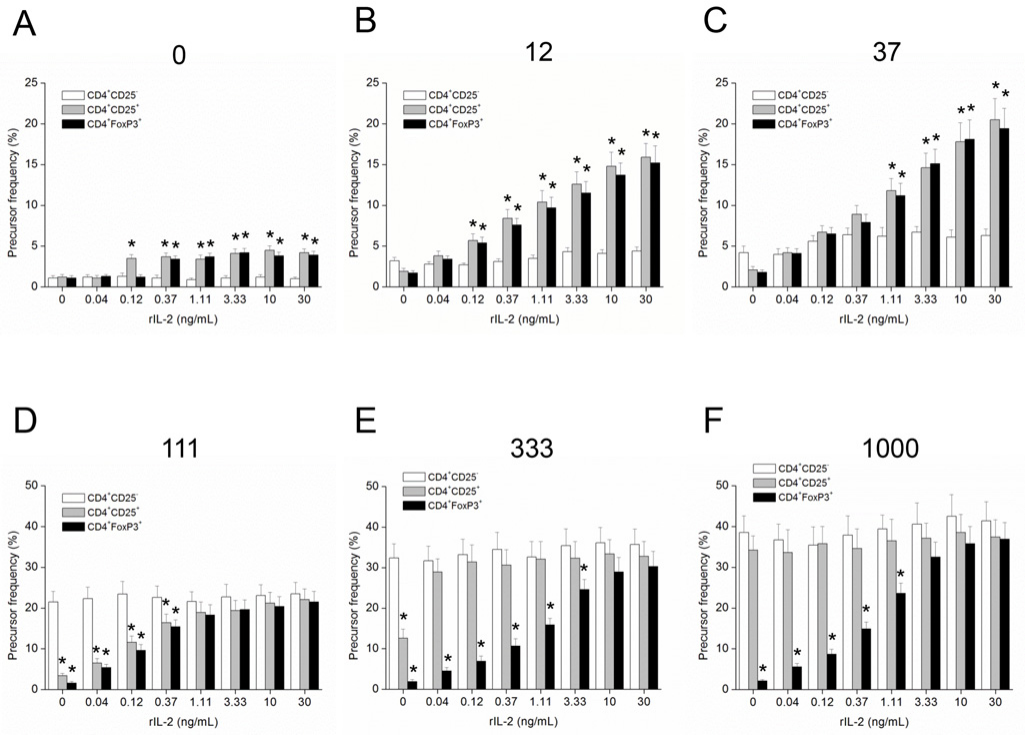
In presence of exogenous IL-2, CD4^+^CD25^+^ and CD4^+^FoxP3^+^ cells were more sensitive than CD4^+^CD25^-^ responder cells in a proliferative response *in vitro*. CFSE-labeled CD4^+^CD25^+^, CD4^+^FoxP3^+^ or CD4^+^CD25^-^ responder cells were stimulated with varying amounts of immobilized anti-CD3e (numbers in ng/well) plus soluble anti-CD28 (100 ng/mL) in the presence of varying concentrations of exogenous IL-2 in an APC-free system. Data are mean ± SE of three separate experiments with 4 replicate wells per dilution. *, P<0.05, between CD4^+^CD25^+^ or CD4^+^FoxP3^+^ fraction and CD4^+^CD25^-^ responder cells.

In order to investigate and compare the proliferative responsiveness of CD4^+^CD25^-^, CD4^+^CD25^+^ and CD4^+^FoxP3^+^ cells in a more defined fashion, we stimulated the cells in separate cultures of an APC-free system in the presence of various concentrations of exogenous IL-2. In the absence of APCs, a stronger stimulation is necessary for T cell proliferation; as such, the cells were stimulated with various amounts of immobilized anti-CD3e plus soluble anti-CD28 (100 ng/mL). In response to a weaker stimulation (12 and 37 ng/well of immobilized anti-CD3e), the proliferative responses of CD4^+^CD25^+^ and CD4^+^FoxP3^+^ cells were higher than that of CD4^+^CD25^-^ responder cells and were exquisitely dependent on the concentrations of added IL-2 (Fig 2B and 2C). On the other hand, in response to relatively strong stimulation (111 to 1,000 ng/well of immobilized anti-CD3e), the proliferative response of CD4^+^CD25^-^ responder cells was higher than those of CD4^+^CD25^+^ and CD4^+^FoxP3^+^ cells, but was not dependent on the concentrations of exogenous IL-2 (Fig 2D, 2E and 2F). In contrast, CD4^+^FoxP3^+^ cells were dependent on the concentrations of exogenous IL-2 in the proliferative response, irrespective of the strength of stimulation. Meanwhile, CD4^+^CD25^+^ cells were dependent on exogenous IL-2 in the proliferative response in the range of a weaker stimulation (12 to 111 ng/well of immobilized anti-CD3e), but not in the range of a stronger stimulation (333 to 1,000 ng/well of immobilized anti-CD3e).

### Low dose IL-2 enhanced, while high dose abrogated, the suppressive activity of CD4^+^CD25^+^ and CD4^+^FoxP3^+^ cells

**Fig 3:**
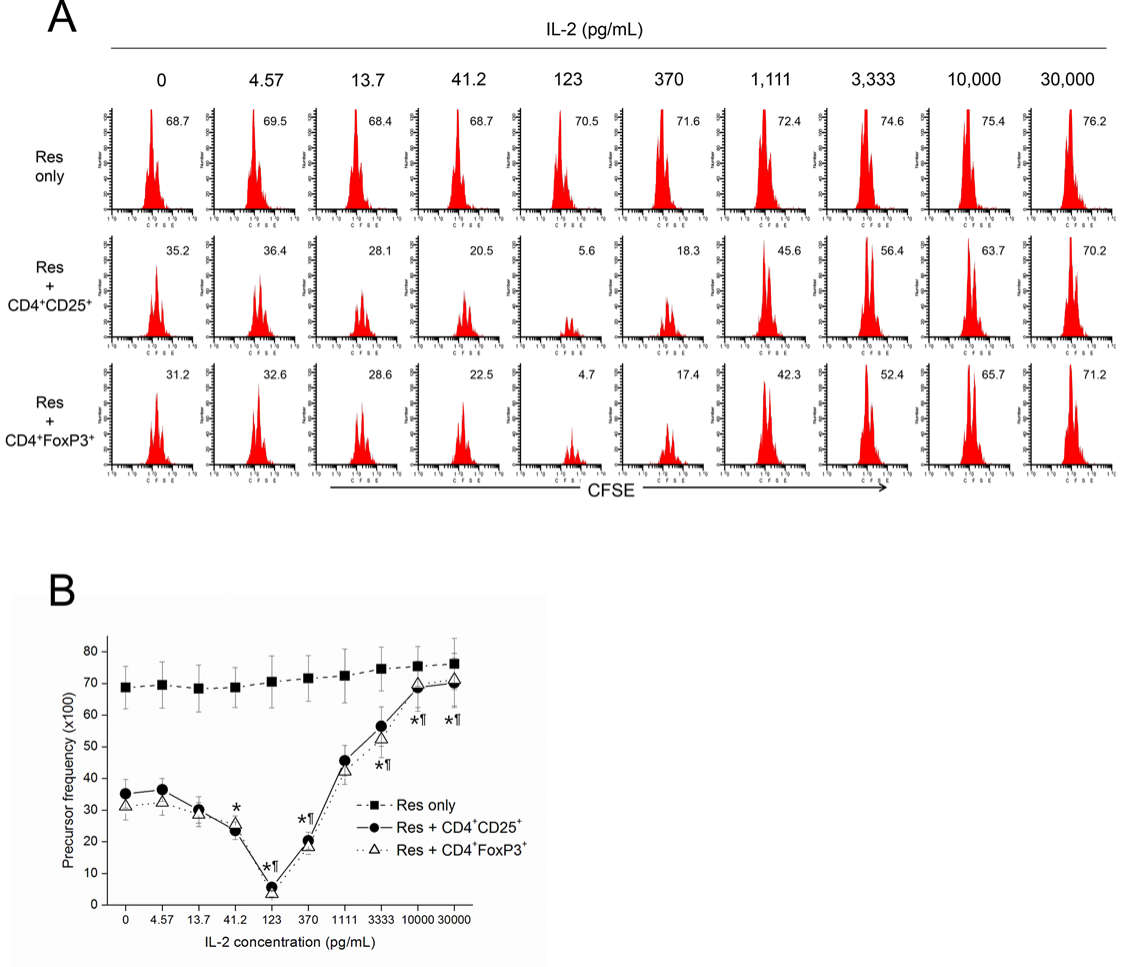
Low dose IL-2 enhanced, while high dose abrogated the suppressive activity of Tregs. CFSE-labeled CD4^+^CD25^-^ responder cells were cultured in the absence or presence of Tregs (CD4^+^CD25^+^ or CD4^+^FoxP3^+^ cells) to study the suppressive activity of Tregs. Responder cells, Tregs and CD11c^+^ dendritic cells were cultured together at a ratio of 1:0.5:0.2, respectively, with stimulation by soluble anti-CD3e (333 ng/mL). After three days of co-culture, the cells were harvested and stained with CD4-Pacific blue, and whole cells were acquired for analysis. Live CD4^+^CFSE^+^ cells were gated for analysis of the proliferative response of CFSE-labeled responder cells. A representative series of histograms from three separate experiments with the same pattern of results are shown (A). The numbers are precursor frequency (Pf) (%). Data are mean ± SE of three separate experiments with 4 replicate wells per dilution (B and C). P<0.05, compared with the control values (in the absence of IL-2) of CD4^+^CD25^+^ cells (*) or CD4^+^FoxP3^+^ cells (¶).

Previously, it has been reported that exogenous IL-2 abrogated the suppressive activity of CD4^+^CD25^+^ Tregs *in vitro* [18,35]. Meanwhile, we observed that CD4^+^CD25^+^ and CD4^+^FoxP3^+^ cells were responsive to very low concentrations of exogenous IL-2 even in presence of weak anti-CD3 stimulation signals (Fig 2). This may be due to the relatively high density of CD25 on Tregs, and that Tregs are able to sense a large range of IL-2 in their environment. We speculated there might be a range of IL-2 concentration that works selectively on Tregs. To this end, we quantitatively analyzed the suppressive activity of CD4^+^CD25^+^ and CD4^+^FoxP3^+^ cells in the presence of relatively low concentrations of exogenous IL-2 (Fig 3). As reported previously, higher concentrations of IL-2 (greater than 2 ng/mL) abrogated the suppressive activity of CD4^+^CD25^+^ and CD4^+^FoxP3^+^ cells. By contrast, very low concentrations of IL-2 (about 0.03 to 0.3 ng/mL) enhanced the suppressive activity of CD4^+^CD25^+^ and CD4^+^FoxP3^+^ cells. This demonstrated a bimodal response of both of these cell types to exogenous IL-2. Meanwhile, the CD4^+^CD25^-^ responder cells in the absence of Tregs were almost insensitive to the exogenous IL-2 in the proliferative responsiveness.

## Discussion

For a long time, many investigators believed that Tregs do not proliferate well *in vitro* [36]. On the other hand, an active proliferative response from Tregs comparable to that from Teffs was observed *in vivo* [37,38,39]. This suggested different behaviors of Tregs being proliferative *in vivo* but hypo-proliferative *in vitro* [6]. In the present study, we demonstrated that if exogenous IL-2 was present Tregs proliferated well *in vitro* (Fig 2). Dependency on exogenous IL-2 is an intrinsic property of Tregs endowed by expression of Foxp3 as it suppresses transcription of IL-2 while activating the transcription of IL-2Rα (CD25) via AML1/Runx1 transcription factor [16]. As a result, Treg proliferation is dependent on the amount of IL-2 provided by Teffs, with IL-2 reflecting the activity of Teffs [10,17,19,40]. Thus Tregs proliferate and suppress according to the activity of their target cells forming an elegant feedback loop of regulation [41].

In the present study, in the absence of exogenous IL-2, pure CD4^+^FoxP3^+^ cells did not proliferate even in response to a strong stimulation with costimulatory anti-CD28 (Figs 1 & 2). On the other hand, CD4^+^CD25^+^ cells proliferated well *in vitro* in response to strong stimulation. It seems contradictory to the intrinsic dependency of Tregs on exogenous IL-2, but the minor FoxP3^-^ fraction in the CD4^+^CD25^+^ cells (11.5 ± 1.3% of the total) (S1 Fig) might secrete sufficient IL-2 that is adequate for the proliferation of remaining FoxP3^+^ cells in the fraction in response to a strong stimulation. We measured the concentrations of IL-2 in the supernatants of CD4^+^CD25^+^ Tregs post activation and they ranged from 50 to 100 pg/mL on the 1^st^ day after a strong stimulation [41]. These relatively low concentrations of IL-2 are sufficient to support the proliferation of Tregs as we have shown relatively low concentrations of exogenous IL-2 enhanced the proliferation of Tregs even in response to a weak stimulation (Fig 2B & 2C). In contrast, exogenous IL-2 enhanced the proliferative response of CD4^+^CD25^-^ responder cells only a little in response to very weak stimulation (Fig 2B & 2C), while CD4^+^CD25^-^ responder cells were almost insensitive to exogenous IL-2 in their proliferative responsiveness in response to relatively strong stimulation (Fig 2E & 2F and Figs 3A & 2B), suggesting autocrine fashion in the supply of IL-2.

In the co-cultures of Tregs and the responder cells in the absence of exogenous IL-2, both the CD4^+^CD25^+^ and CD4^+^FoxP3^+^ cells proliferated well even in response to weak stimulation and without a costimulatory signal from anti-CD28. This suggested secretion of IL-2 by the responder cells although their proliferation was significantly suppressed by the Tregs. Actually we measured the concentrations of IL-2 in the culture supernatants on the 1^st^ day after stimulation and low but detectable concentrations of IL-2 were present [41]. Taken together, we conjecture this IL-2 was from the responder cells right after stimulation, but thereafter their proliferation was suppressed by Tregs.

Our meticulous investigation of the proliferative responsiveness of Tregs in comparison with the responder cells in separate cultures of an APC-free system showed that Tregs were more sensitive than the responder cells in the proliferative responsiveness (Fig 2). Responsiveness sensitivity may be related to the functional sensitivity of Tregs previously documented by Sakaguchi and co-workers: they reported that Tregs suppressed Teffs even in 100-times lower concentration of activating antigen that is necessary to induce activation of Teffs [35]. Taken together, we propose that Tregs are more sensitive to activation than Teffs as reflected in both proliferation and function.

A characterization of Tregs’ sensitivity to antigen and various cytokine signals may help in development of more successful clinical procedures involving Tregs. Subcutaneous immunotherapy (SCIT) has been empirically applied for more than a 100 years, but only recently, Tregs were noted to play a role as Treg expansion was observed following SCIT-led desensitization to specific venom and hay fever [42,43]. We still do not fully understand of why Tregs expand during SCIT. Our results may explain this as a picture of weak stimulation during the initial stage of immunotherapy favoring Treg expansion as these cells are more sensitive to low antigen levels.

Previously, it was reported that exogenous IL-2 added to a co-culture of Tregs and responder cells abrogated the suppressive activities of Tregs [18,35]. These *in vitro* data are compatible with the *in vivo* observations that IL-2 administration resulted in autoimmunity or augmented immune responsiveness [24,25,26,27], but they cannot explain an opposing observation such as IL-2 immunotherapy preventing and reversing IDDM in NOD mouse model [28]. In our opinion, as IL-2 is central to a feedback regulatory circuit, with levels of IL-2R being different between Tregs and Teffs, the concentration of IL-2 is critical to the fate of these two kinds of cells. In addition, IL-2R signaling is distinctive between Tregs and Teffs, and the signaling threshold is lower in Tregs than Teffs [44,45]. Thus, there might be a range of low concentrations of IL-2 selectively stimulating Tregs. In the present study, Treg suppressive function was maximal around 123 pg/mL (41.2 ~ 370 pg/mL). In contrast, high concentrations of IL-2 may saturate IL-2R on Tregs and bind to IL-2R on Teffs as well. The suppressive mechanism of Tregs also involve IL-2 signaling in several ways, including capturing and depriving IL-2 of Teffs by highly expressed IL-2Rs on Tregs, suppressing IL-2 transcription in Teffs and inducing early shedding of IL-2Rs from Teffs [8,20,41]. Accordingly, high concentrations of exogenous IL-2 may directly abolish the suppressive function of Tregs. In the present study, Treg suppressive function was abrogated in the presence of exogenous IL-2 equal to or more than 3.3 ng/mL.

Thus, IL-2 may be perfectly suited as a master switch between these two cell types as it is secreted rather quickly from activated T cells and its action is important for early stages of T cell response [46]. The concentration of exogenous IL-2 that abrogated the suppressive activity of Tregs *in vitro* reported by de la Rosa et al was 30 ng/mL, which was far higher than needed to support the proliferation of Tregs per our experiments [18]. We expected and observed that lower concentrations of exogenous IL-2 actually enhanced rather than lowered the suppressive activity of Tregs (Fig 3).

The various functional roles attributed to IL-2 have been changing [47]. Initially, it was discovered as a T cell growth factor (labeled as TCGF) [48]. However, that concept of a TCGF changed with observation that a genetic deficiency of IL-2 or IL-2R resulted inflammatory bowel disease (IBD), an overactive immune system situation [49]. A new concept of IL-2 was then promoted making it a death-promoting factor that induces activation-induced cell death of T cells [50]. After the discovery of IL-15 and Tregs the concept on IL-2 changed again: It was thought that IL-2 was not essential to activate T cells as IL-15 could replace it [51], but that IL-2 was essential for the survival and function of Tregs [13,52]. At the moment, the role IL-2 as being the key cytokine for the survival and function of Tregs is prevalent and this is supported by our data [53]. In addition, based on our observations, we would like to propose that the concentration of IL-2 is critical to the modulation of regulatory circuit operated by Tregs.

IL-2 has shown promise for the treatment of a number of cancer types (melanoma, renal cell carcinoma, hepatocellular carcinoma, mesothelioma, ovarian cancer, gastrointestinal cancer, lung cancer, and basal cell carcinoma) [23,24,54]. IL-2 has also shown promise for treatment of a number of autoimmune and inflammatory diseases. In particular, low dose IL-2 administration is under active clinical investigation for the therapeutic effects in type 1 diabetes, hepatitis C virus-induced vasculitis, alopecia areata, systemic lupus erythematosus and chronic graft versus host disease [9,55,56].

IL-2 signaling can also be modulated by an administration of anti-IL-2 or anti-IL-2R antibody. An anti-CD25 is currently being used for prevention of allograft rejection after kidney transplantation [57]. Autoimmune diseases and a variety of lymphoid neoplasms are also investigated for the therapeutic application of an anti-CD25 antibody as the list of diseases under consideration for a therapeutic modulation of IL-2 signaling is expanding [54]. We believe that an in-depth study of regulation of Tregs by IL-2, such as our work, will contribute to the improvement of clinical outcomes for immunotherapy using IL-2 or an anti-IL-2R antibody.

In summary, we observed that Tregs proliferated well *in vitro* and that Tregs were more sensitive than the responder cells in their proliferative response. We also observed that the concentration of IL-2 was critical in regulating the suppressive function of Tregs. We hope our observations may contribute to a better understanding of the regulation of Tregs suppressive activity and the role IL-2 plays in this parameter of their function.

## Supporting Information

S1 Fig Overlapping distribution of CD4^+^CD25^+^ and CD4^+^FoxP3^+^ fractions. Flow cytometric analysis of the splenocytes prepared from four Foxp3 knock-in mice by lysis of erythrocytes showed that 2.6 ± 0.4% were CD4^+^CD25^+^ and 2.8 ± 0.3% were CD4^+^FoxP3^+^, respectively. In the CD4^+^CD25^+^ population, 88.4 ± 3.5% were FoxP3^+^, while 11.5 ± 1.3% were FoxP3^-^. Of the CD4^+^FoxP3^+^ cells, 74.6 ± 4.2% were CD4^+^CD25^+^, while 22.5 ± 2.2% were CD4^+^CD25^-^. Data are mean ± SE of three separate experiments with four mice analyzed separately.
(TIF)
