# Supplementary figures and images for: Functional Modulation of Regulatory T Cells by IL-2
Source: PLoS One. 2015 Nov 3;10(11):e0141864. doi: 10.1371/journal.pone.0141864 (PMC4631326; doi:10.1371/journal.pone.0141864)

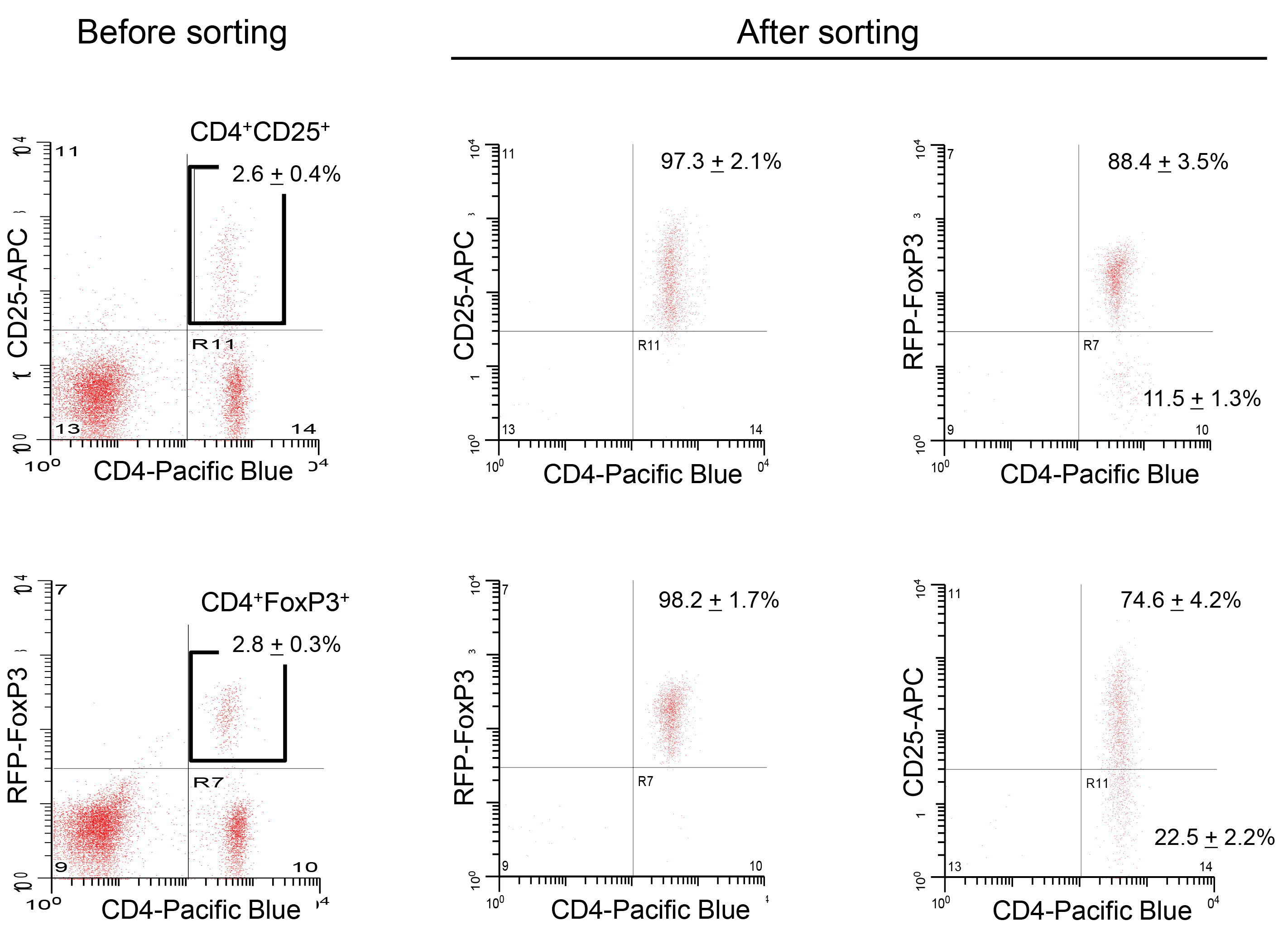

Supplement: S1 Fig — Flow cytometric analysis of the splenocytes prepared from four Foxp3 knock-in mice by lysis of erythrocytes showed that 2.6 ± 0.4% were CD4+CD25+ and 2.8 ± 0.3% were CD4+FoxP3+, respectively. In the CD4+CD25+ population, 88.4 ± 3.5% were FoxP3+, while 11.5 ± 1.3% were FoxP3-. Of the CD4+FoxP3+ cells, 74.6 ± 4.2% were CD4+CD25+, while 22.5 ± 2.2% were CD4+CD25-. Data are mean ± SE of three separate experiments with four mice analyzed separately. (TIF) [file pone.0141864.s001.tif]
